# Malignant mesothelioma associated with localized myocardial fibrosis: a case report

**DOI:** 10.1186/s12872-021-02079-2

**Published:** 2021-06-07

**Authors:** Kristyna Vymetalova, Pavol Tomasov, Rostislav Polasek

**Affiliations:** 1 Department of Cardiology, Liberec Regional Hospital, Husova 357/10, 460 63 Liberec, Czech Republic; 2 Faculty of Health Studies, Technical University of Liberec, Liberec, Czech Republic

**Keywords:** Case report, Heart failure, Malignant mesothelioma, Cardiac magnetic resonance

## Abstract

Left ventricular dysfunction is a common reason for patients’ referral to cardiology departments for examination. Cardiac involvement is one of the possible yet rare presentations of malignant mesothelioma. We present a case of a patient in whom a routine cardiac examination and imaging revealed malignant mesothelioma. We discuss a possible association between a malignant tumor and myocardial scarring and how the oncologic treatment is influenced by concomitant heart failure. This article aims to raise awareness of the importance of multidisciplinary cooperation and thinking beyond the daily routine of our specialty to ensure the quality care of our patients. It also forced us to think about the possible causes of the association between malignant mesothelioma and myocardial fibrosis.

**Supplementary Information:**

The online version contains supplementary material available at 10.1186/s12872-021-02079-2.

A 45-year-old man was referred to our cardiology department with pleuritic chest pain without any symptoms of heart failure. Transthoracic echocardiography (TTE) revealed moderate systolic dysfunction of the left ventricle (LV) due to wall motion abnormalities of lateral and posterobasal segments of the LV associated with localized pericardial effusion which was first suspected to be a pseudoaneurysm. Ejection fraction of the LV was approximately 35%. The systolic function of the right ventricle was normal and both the atria were not dilated. Ventriculography revealed dyskinesia of the basal segments of the lateral and posterior walls and no signs of extravasation of the contrast agent. Surprisingly, there was no significant coronary artery disease on coronary angiography explaining the findings above.

We performed cardiac magnetic resonance (CMR) imaging which confirmed moderate systolic dysfunction of the LV and nonischemic pattern of non-homogenous late contrast agent enhancement of the thin lateral and posterior walls without myocardial edema (Fig. 1, Additional file 1: Movie File 1). The lateral wall of the LV was 3 mm thin compared to the interventricular septum which was 12 mm thick. The LV was not dilated with an end-diastolic volume of 159 ml. The aforementioned pericardial effusion was not apparent, instead, CMR raised suspicion of pleural malignant mesothelioma adjacent to the affected myocardium.

**Fig. 1:**
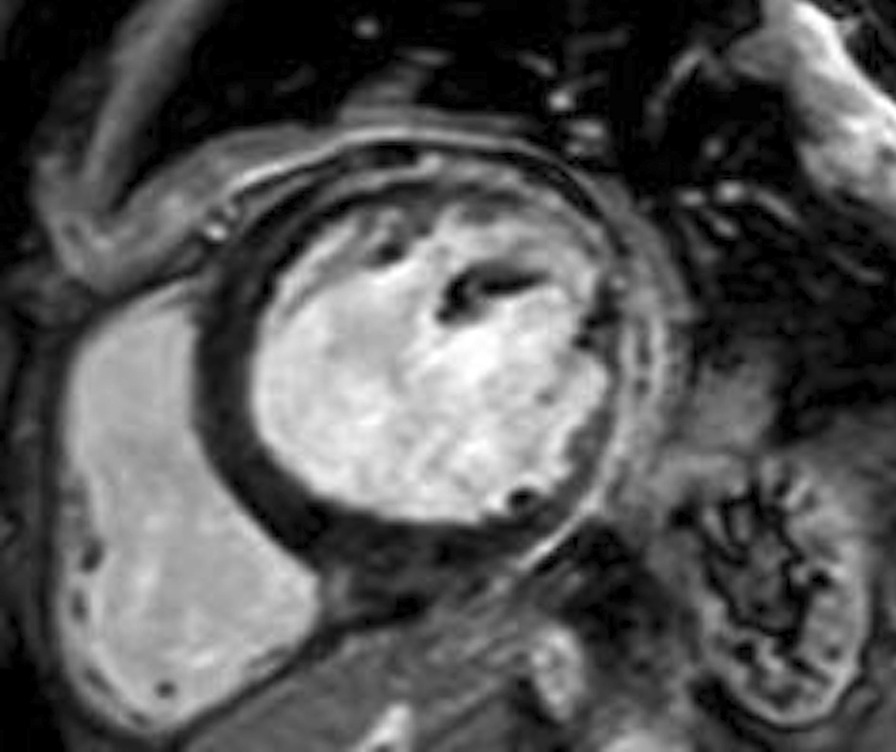
This gadolinium-enhanced cardiac magnetic resonance image shows non-homogenous late contrast agent enhancement of the thin posterolateral wall of the left ventricle. It also revealed thickening of the pleura suggestive of malignant mesothelioma

This diagnosis was subsequently verified by computed tomography (CT) imaging (Fig. 2) and confirmed with transparietal CT navigated biopsy of the pleura. Positron emission tomography (PET) was performed finding fluorodeoxyglucose (FDG) avid thickening of the pleura in close proximity to the pericardium. No significant invasion into the surrounding structures or the chest wall was apparent. The patient was referred to the oncology department to advise on further management. Detailed history did not reveal any asbestos or radiation exposure.

**Fig. 2:**
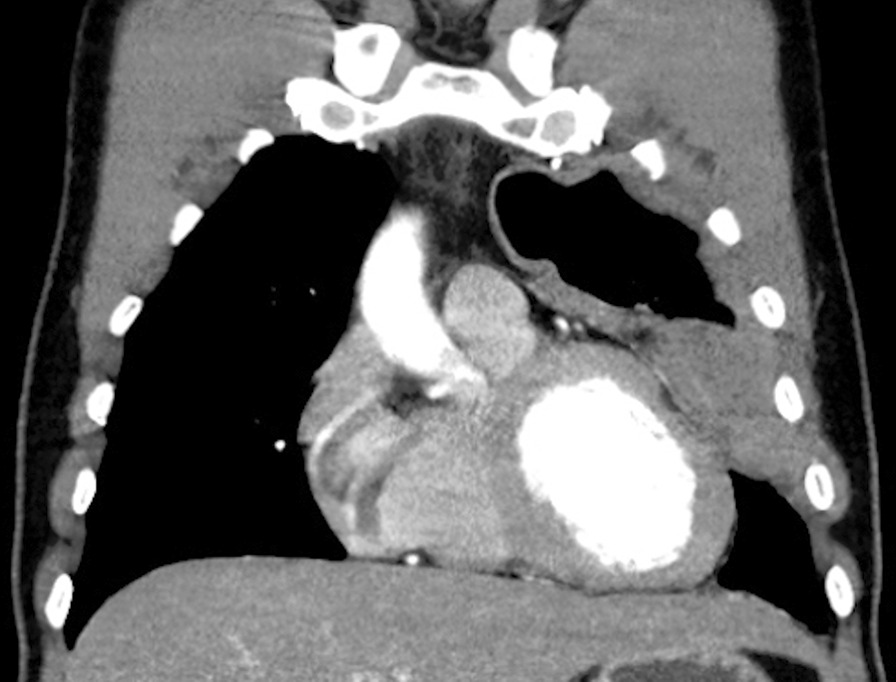
The computed tomography imaging scan shows a tumor mass adjacent to the lateral wall of the left ventricle. This image demonstrates proximity of the tumor mass to the affected myocardium

While medical therapy for heart failure with telmisartan, bisoprolol, and spironolactone was initiated, dysfunction of the LV persisted and the patient remained asymptomatic. After 4 cycles of lower-dose less-toxic neoadjuvant chemotherapy with carboplatin and pemetrexed, the patient underwent extrapleural pneumonectomy with resection of the diaphragm and pericardium and subsequent hyperthermic intrathoracic chemotherapy with cisplatin. There was a need for inotropic support and blood transfusion shortly after the surgery, uncomplicated weaning led to extubation the fourth post-operative day. The chest drain had to be reinserted due to development of tension fluidothorax. The patient developed signs of heart failure which was managed with diuretics shortly thereafter. He was discharged the twentieth post-operative day and was recommended for active surveillance as he did not qualify for adjuvant chemotherapy or radiotherapy due to LV dysfunction. Histopathology revealed tumor free margins of the resected material, therefore, no direct invasion of the tumor into the myocardium was expected, leaving the localized fibrosis of the LV unexplained.

After the surgery, systolic function of the LV deteriorated and the patient tolerated only very low doses of medical therapy for heart failure due to symptomatic hypotension. LV dysfunction thus led to reducing the dose of neoadjuvant chemotherapy; moreover, it made our patient ineligible to receive any adjuvant treatment. Follow-up PET revealed FDG avid tissue in the site of the resected tumor. It could not be determined whether it represents just postoperative fibrosis or is a sign of recurrence of the tumor. One year after the surgical treatment, echocardiography revealed severe LV systolic dysfunction, ejection fraction was approximately 20–25%, and dysfunction of the right ventricle which was not associated with any significant increase in pulmonary arterial pressure. The patient is in functional class NYHA (New York Heart Association) III and was able to return to his job despite significant exertion intolerance. Given the worsening systolic dysfunction of the LV, dysfunction of the right ventricle and symptoms of heart failure, the patient will be referred to assess his eligibility for a biventricular assist device.

## Discussion

Malignant mesothelioma is a rare malignant disease affecting the pleura, less commonly the pericardium and other serous membranes. The most common presenting symptoms are dyspnea, cough, and chest pain. Concerning cardiac involvement, malignant mesothelioma of the pericardium is known to cause constrictive pericarditis and pericardial effusion [1]. Invasion of the tumor into the myocardium causing LV dysfunction has also been described [2].

We searched medical databases for keywords “mesothelioma left ventricular dysfunction”/scarring”/aneurysm”, “mesothelioma heart failure”, and reviewed available case reports. To our knowledge, a case of malignant mesothelioma causing localized LV dysfunction has not yet been described apart from cases in which malignant mesothelioma invaded the myocardium [3]. Since an invasion of the tumor into the myocardium was not detected in our patient, we attributed formation of a myocardial scar to proximity of malignant mesothelioma with the myocardium. External compression by the tumor mass is one of the possible causes of localized LV dysfunction. Another possible yet unexplored mechanism is a release of the cytokines and other mediators from malignant mesothelioma to the adjacent myocardium.

In general, tumors and other masses can cause LV dysfunction by external compression of the coronary artery [4, 5]. In our patient, this mechanism is unlikely in the absence of correlating changes in the coronary angiography. Mechanical compression of the myocardium is also a possible explanation for signs and symptoms of heart failure as in patients with pectus excavatum [6]. Also, a case of malignant mesothelioma causing heart failure due to mechanical compression of right atrium has been described [7]. However, it is unlikely to cause localized dysfunction of the LV and scar formation.

Malignant mesothelioma is known to be resistant to cytostatic therapy and the immune suppressive microenvironment is likely contributing to this therapy resistance. Therefore, it was studied in an attempt to target the cytokines and their receptors deemed responsible for the progression of the tumor with the biological therapy. Malignant mesothelioma was found to attract cancer-associated fibroblasts and fibrocytes and macrophages with their collagenolytic activity responsible for tissue remodeling [8]. In our patient, these mechanisms may have also influenced the myocardium in close proximity to the tumor causing its fibrosis.

While we cannot omit the possibility that the correlation of localization of mesothelioma and the myocardial scar is coincidental, such a perfect correlation of the site of the tumor and myocardial fibrosis is striking. Apart from that, localized myocarditis is likewise improbable in this case as CMR did not reveal any myocardial edema. Both malignancy and myocarditis could be caused by radiation, however, our patient did not have any history of radiation exposure.

## Conclusion

This case taught us to think beyond the daily routine of treating patients with localized dysfunction of the LV. In the era of superspecialization in medicine, multidisciplinary cooperation is more important than ever for the quality of patient care. In cases like this, the therapeutic interests of one specialty may influence the intentions of others' which we need to bear in mind treating patients with similarly complex diagnoses. It also forced us to think about the possible causes of the association between malignant mesothelioma and myocardial fibrosis.

## Supplementary Information

**Additional file 1: Movie File 1**. This movie file demonstrates the cardiac motion in a short-axis cine loop. It re-veals akinesia of the posterolateral wall of the left ventricle.

## Data Availability

Not applicable.

## Abbreviations

TTE: Transthoracic echocardiography
LV: Left ventricle
CMR: Cardiac magnetic resonance
CT: Computed tomography
FDG: Fluorodeoxyglucose
NYHA: New York Heart Association

## References

[1] Fernandes R, Nosib S, Thomson D, Baniak N (2014). A rare cause of heart failure with preserved ejection fraction: primary pericardial mesothelioma masquerading as pericardial constriction. BMJ Case Rep.

[2] Wadler S, Chahinian P, Slater W, Goldman M, Mendelson D, Holland JF (1986). Cardiac abnormalities in patients with diffuse malignant pleural mesothelioma. Cancer.

[3] Barroso AS, Leite S, Friões F (2017). Pericardial mesothelioma presenting as a suspected ST-elevation myocardial infarction. Rev Port Cardiol.

[4] Demerouti E, Petrou E, Karatasakis G, Mastorakou I, Athanassopoulos G (2015). First application of coronary flow reserve measurement for the assessment of left main compression syndrome in pulmonary hypertension. Can J Cardiol.

[5] Yassin NY, Tomasov P, Horak J, Polasek R (2019). Occlusion of epicardial coronary arteries by localized pericardial calcification. J Am Coll Cardiol Case Rep.

[6] Lollert A, Emrich T, Eichstädt J, Kampmann C, Abu-Tair T, Turial S (2017). Differences in myocardial strain between pectus excavatum patients and healthy subjects assessed by cardiac MRI: a pilot study. Eur Radiol.

[7] Lisowska A, Knapp M, Sobkowicz B, Musiał WJ (2005). Severe right-ventricular heart failure due to malignant pericardial mesothelioma. Kardiol Pol.

[8] Chu GJ, van Zandwijk N, Rasko JEJ (2019). The immune microenvironment in mesothelioma: mechanisms of resistance to immunotherapy. Front Oncol.

